# Exogenous pyruvate is therapeutic against colitis by targeting cytosolic phospholipase A2

**DOI:** 10.1016/j.gendis.2025.101571

**Published:** 2025-02-22

**Authors:** Sadaf Hasan, Nabil Ghani, Xiangli Zhao, Julia Good, Chuan-ju Liu

**Affiliations:** a Department of Orthopedic Surgery, New York University Grossman School of Medicine, New York, NY 10010, USA; b Department of Medicine, Division of Internal Medicine, Saint Peter's University Hospital, Rutgers University, New Brunswick, NJ 08901, USA; c Department of Cell Biology, New York University Grossman School of Medicine, New York, NY 10010, USA; d Department of Orthopedics & Rehabilitation, Yale School of Medicine, New Haven, CT 06510, USA

**Keywords:** Colitis, Cytosolic phospholipase A2, Drug affinity-responsive target stability assay, Inflammation, Pyruvate, TNFα/NFκB signaling

## Abstract

Ulcerative colitis is an idiopathic, chronic inflammatory bowel disease. Its pathogenesis is multifactorial involving inflammation and immune dysregulation. Proinflammatory TNFα/NFκB signaling is believed to play a cardinal role in ulcerative colitis. Growing evidence indicates the molecular interactions between the cellular metabolites and different phases of inflammation. This study aims to identify the metabolites that can inhibit TNFα/NFκB signaling and are potentially therapeutic against various TNFα-associated inflammatory diseases, particularly inflammatory bowel diseases. We performed *in vitro* and *in vivo* screening of cellular metabolites to inhibit TNFα/NFκB signaling. Multiple confirmation assays, including NFκB translocation, quantitative real-time PCR, ELISA, immunofluorescence staining, and RNA sequencing analysis were executed. Drug affinity-responsive target stability assay with proteomics was utilized for target identification. cPLA2 ablated mice with dextran sodium sulfate-induced colitis were employed to assess pyruvate's dependence on its molecular target in attenuating ulcerative colitis pathogenesis. Metabolite screening and subsequent validation with multiple approaches led to the isolation of pyruvate, a glycolytic metabolite, and a critical node in several metabolic pathways, as a novel inhibitor of TNFα/NFκB signaling. Importantly, pyruvate suppressed inflammation, preserved colonic histology, maintained tight junction proteins, and regulated permeability in the ulcerative colitis model. Additionally, cPLA2 was identified as a previously unknown target of pyruvate and pyruvate largely lost its therapeutic effects against ulcerative colitis in cPLA2-deficient mice. Conclusively, this study not only unveils pyruvate as an antagonist of TNFα/NFκB signaling and therapeutic intervention against colitis but also provides mechanistic insight into the mode of action of pyruvate.

## Introduction

Ulcerative colitis (UC) has been recognized as one of the main types of inflammatory bowel disease (IBD).^1^, ^2^, ^3^ IBD is a chronic intestinal inflammatory disorder that is presented by abdominal pain, bloody mucopurulent stool, diarrhea, fatigue, and weight loss.^3^ Despite the obscure pathogenesis of IBD, growing evidence suggests that it is an inappropriate inflammatory response against enteric microorganisms in a genetically vulnerable host.^4^^,^^5^ Among these pathological processes, the disruption of the gut epithelial junction and altered expression of tight junction proteins, including claudins, occludin, and zonula occludens, which results in increased intestinal permeability, is thought to mark an early event in IBD etiology.^6^ This event allows enteric bacteria to infiltrate through a barrier leak, triggering an exaggerated immune response and severe colonic inflammation.^7^, ^8^, ^9^, ^10^

Because of these intricate etiologic factors, various murine models of chronic intestinal inflammation have been developed to elucidate the pathogenesis of IBD.^11^ These models have become indispensable tools in providing significant insights into the immunological, morphological, and histopathological mechanisms underlying chronic mucosal inflammation.^10^^,^^12^ Among these, dextran sodium sulfate (DSS)-induced colitis is the most broadly used experimental model to induce epithelial damage,^13^ as it offers simplicity,^14^ rapidity (the onset and induction of inflammation are immediate), controllability (chronic or acute models of intestinal inflammation can be attained by altering DSS concentration and its frequency of administration),^15^^,^^16^ as well as reproducibility while replicating pathologies like cytokine dysregulation in human UC.^17^ Therefore, they are powerful tools to investigate the facilitators of the innate, adaptive, and regulatory immune response of the intestines.^14^^,^^17^

Numerous studies have demonstrated that the tumor necrosis factor-alpha (TNFα)/nuclear factor-kappa B (NFκB) pathway plays a central role in the pathogenesis of UC through cytokine release via modulating the inflammatory and immune response.^18^, ^19^, ^20^ It is established that NFκB is highly expressed in the intestinal mucosal epithelium as well as in cryptepithelial cells in UC. Moreover, the expression of nuclear NFκB is substantially higher than cytoplasmic NFκB, marking its nuclear translocation under an inflammatory state.^21^ This translocation results in the production of pro-inflammatory cytokines in lamina propria mononuclear cells contributing to the pathogenesis of UC.^22^ Hence, one of the most crucial strategies in the treatment of UC focuses on maintaining intestinal mucosal barrier function and the regulation of the NFκB pathway.^3^^,^^18^

Currently, the drugs that are being used for the management of UC include aminosalicylic acids, adrenal glucocorticoids, and immunosuppressants. Most of them act by impeding the metabolism and immune response and thus, their long-term use has been associated with adverse reactions including severe toxicity.^23^^,^^24^ Furthermore, the anti-TNFα agents that undoubtedly improved the management of UC were at the disadvantage of not being available as oral formulations.^25^^,^^26^ Therefore, it is desirable to explore new orally available small molecules and their elucidated mechanism in the treatment of UC. Consequently, present research intensely focuses on assessing several metabolites owing to their association with intestinal inflammation and IBD pathogenesis.^27^ Prominent among these are α-ketoglutarate,^28^ tryptophan,^29^ and leucine.^30^

Endogenous pyruvate is a pivotal biochemical intermediate produced by glucose via glycolysis. It occupies a crucial metabolic node by virtue of its position at the intersection of glycolysis and the tricarboxylic acid cycle.^31^ However, exogenous pyruvate is integrated into the cells via mono-carboxylate transporters.^32^ Many shreds of evidence support that pyruvate is not merely an inert byproduct of metabolism but shows systemic therapeutic effects and predominantly functions as an antioxidant.^33^ Treatment with exogenous pyruvate has been shown to ameliorate hyperglycemia,^34^ retinopathy,^35^ and nephropathy in streptozotocin-induced diabetic animals.^39^ Additionally, many pieces of evidence support its pharmacological effect in improving cardiac function after coronary ischemia and critical medical conditions, like severe sepsis, acute respiratory distress syndrome, burn injury, acute pancreatitis, and stroke^36^ and its supplementation for the treatment of osteoporosis in postmenopausal women demonstrated good tolerability and bioavailability.^37^ In contrast, the disruption of the targeted gene of murine pyruvate kinase, which catalyzes the conversion of phosphoenolpyruvate to pyruvate, was shown to deteriorate diabetic nephropathy.^38^

Phospholipids are the primary component of the lipid bilayer in mammalian cell membranes.^39^ Group IVA cytosolic phospholipase A2 (cPLA2) is an 85 kDa protein that belongs to the PLA2 enzyme superfamily. The six members of the cPLA2 family (α, β, γ, δ, ε, and ζ) demonstrate merely 30 % similarity. They vary in their enzymatic characteristics, tissue distribution, and subcellular locations, indicating that each plays a distinct role.^39^ However, the regulation of cPLA2α activity is complex and involves multiple factors, including phosphorylation, subcellular localization, and protein–protein interactions.^39^ When activated, cPLA2 moves from the cytosol to the membrane, where it catalyzes the hydrolysis of membrane phospholipids. This results in the production of diverse lipid mediators including arachidonic acid. Arachidonic acid then activates NFκB, leading to the release of proinflammatory cytokines.^39^

Nevertheless, the role of pyruvate in UC has limited data, and particularly, the mechanisms underlying various pyruvate-mediated beneficial effects are essentially unknown. Thus, the present study aimed to explore whether pyruvate supplementation had therapeutic effects on murine UC and to elucidate the molecular mechanisms involved. Consequently, we present comprehensive evidence demonstrating that pyruvate could alleviate pathogenic features in DSS-induced murine colitis by inhibiting TNFα/NFκB signaling, maintaining intestinal mucosal barrier function, decreasing pro-inflammatory cytokines, and improving the diseased activity index. Moreover, using a combined approach of novel target identification methods and proteomics, we also identified cPLA2 as a novel target of pyruvate and it is indispensable for pyruvate's therapeutic function.

## Materials and methods

### Ethical approval

All animal studies were managed following institutional guidelines and approved by the Institutional Animal Care and Use Committee of New York University.

### Animals

All mice were housed in the Skirball Animal Facility of New York University Langone Medical Center and were maintained in a temperature-controlled (23 °C) room on a 12 h/12 h light/dark cycle with *ad libitum* access to food and water in a specific pathogen-free environment. Unless stated otherwise, the mice used were male, typically between 8 and 10 weeks of age, and were age-matched for experiments. Genotypes used for the experiment were C57BL/6J background, TNFα-transgenic mice (TNFα-tg), NFκB Luc, and cPLA2. C57BL/6J and NFκB-luciferase reporter (model number 10499) mice were purchased from Jackson Laboratory (Bar Harbor, ME, USA). cPLA2 heterozygous mice were a generous contribution from Dr. Naikui Liu's lab at Indiana University School of Medicine. These mice originated from Joseph V Bonventre's lab at Brigham and Women's Hospital. These cPLA2 mice are on a C57BL/6 and 129 mixed backgrounds. Wild-type littermates (cPLA2α^+/+^) and global cPLA2 KO (cPLA2α^−/−^) mice were generated by crossing heterozygous cPLA2 (cPLA2^+/−^) mice. TNFα-tg mice were generated by transfecting the human TNFα gene to C57BL/6 background mice, which imitates a systemic inflammatory state.^40^

### *In vivo* bioluminescence imaging for NF-κB signaling

NF-κB luciferase reporter mice were evaluated for inflammatory signals via *in vivo* bioluminescence. For screening, colitis induction was performed by adding 3% DSS (*w*/*v*) to sterile drinking water and was given to the mice in the experiment from day(s) 0–5. The treatment with different metabolites, *viz*., dimethyl fumarate, α-ketoglutarate, glucose-6-phosphate, succinate, citrate, malate, acetyl CoA, succinyl CoA, and pyruvate was also started at day 0 using oral gavage. 5-aminosalicylic acids (5-ASA) were used as a positive control^41^ A. Mice were accordingly allotted to different groups (*n* = 6 per group) including the control group (no DSS), DSS group (DSS challenge), positive control group (DSS challenge + 5-ASA), treatment groups (DSS + respective metabolite under screening), and no luciferin group. After 5 days, the DSS water was replaced with normal water, and on the 7th day, an *in vivo* imaging system was performed. Mice were anesthetized with isoflurane and an intraperitoneal injection of 150 mg/kg luciferin was administered. Imaging was performed (after 10 min) every 5–10 min and extended throughout up to 60 min to determine the peak signal. Bioluminescent signals were quantified using a whole animal bioluminescence imaging system (PerkinElmer IVIS® Spectrum) and analysis software (PerkinElmer Living Image®).^42^

### DSS-induced colitis model

The DSS-induced colitis model was established in age-matched 8-to-10-week-old mice via administration of DSS in drinking water. For groups receiving DSS, 3.0 g of DSS (DSS colitis grade 36,000–50,000 M.Wt, MP Biomedicals™) was fully dissolved in 100 mL of sterile water before the bottle was placed in the cage. Animals received 3% DSS for 7 days, after which they were switched to fresh water for 3 days, however, sodium pyruvate (P2256 Sigma) at a low dose (40 mg/kg) or high dose (100 mg/kg), or 5-ASA (50 mg/kg) was orally administered for a total duration of 10 days unless early euthanasia was required. Sterile water was used to make the dilutions of pyruvate. The water level for each cage was recorded daily and the water was fully changed every two days. The disease activity index was assessed by recording the pathological features including stool consistency, rectal bleeding, and weight loss daily. Scoring for stool consistency, and rectal bleeding, a previously published scoring system was used,^43^ which is summarized in Table S1. Also, the food intake and water consumption were measured daily for the entire duration of the study.

### Histopathological assessment of colon and spleen

The colon and spleen were excised on the 10th day from induction (*n* = 5 or 6) and gently rinsed with phosphate-buffered saline (PBS, pH = 7.4). The colon was excised between the ileocaecal junction and the proximal rectum, close to its passage under the pelvisternum. While dissecting the colon, the absence or presence of adhesion was documented. The whole colon was examined comprehensively for gross examination. Then, the colon was sliced longitudinally, washed with water, and extended on a block for the observation of ulcers.^44^ Both excised colon and spleen were placed on a non-absorbent surface and their length was measured with a ruler, in such a manner that the organs were not stretched. The length including the weight of the colon and spleen was recorded as described previously and the morphology was imaged.^45^ Specimens were fixed in 4% paraformaldehyde solution at 4 °C for 24 h, followed by dehydration in a graded ethanol series, and were then embedded in paraffin blocks using an auto processor (Excelsior ES, Thermo Scientific, USA). 6 μm sections were framed on glass slides and stained with (hematoxylin and eosin) H&E for pathological assessments. H&E staining was performed as described: two changes of xylene for 2 min each; two changes of 100% ethanol for 2 min each; one change of 95% ethanol for 2 min; one change of 80% ethanol for 2 min; one change of distilled water for 2 min; hematoxylin solution (3801570, Leica Biosystems) for 2 min; distilled water for 2 min; differentiating solution (3803590, Leica Biosystems) for 2 min; distilled water for 2 min; blue buffer (3802915, Leica Biosystems) for 2 min; distilled water for 2 min; one change of 95% ethanol for 2 min; eosin solution (3801606, Leica Biosystems) for 2 min; one change of 95% ethanol for 2 min; two changes of 100% ethanol 2 min each; and two changes of xylene 2 min each. Digital imaging was done with a Leica DM2500 microscope (Leica Microsystems, Germany) at fixed 100 × magnification. Histological scoring of colon and spleen tissue was assessed by a trained observer who was blinded to the study design, according to a previously published method.^46^^,^^47^ This scoring for the colon was the combination of inflammatory cell infiltration (microscopic) and intestinal wall (macroscopic) structure integrity where higher scores indicated severer inflammation. These macroscopic and microscopic scores are summarized in Tables S2 and 3. A part of the colon was snap-frozen for mRNA and protein isolation consistently being the same area for each evaluation.

### Blood collection

Blood samples were collected by the previously described method,^47^ followed by serum collection for different biochemical analyses.

### Mouse genomic DNA isolation

Mouse tails of size ∼4 mm were snipped and collected in sterile 1.5 mL microcentrifuge tubes. It was followed by the addition of 100 μL of a tissue digestion buffer (NaOH 25 mM and EDTA 0.2 mM) to each sample. These samples were then heated at 100 °C in a heating block (Isotemp, Fisher Scientific) for 1 h. After thermal digestion, neutralization buffer (100 μL of 40 mM Tris buffer, pH 7.5) was added to each tube. The tubes were then centrifuged for pelleting tail debris at 14,000 *g* for 10 min. The genomic DNA concentration in the supernatant was measured using a spectrophotometer (Nanodrop 200 C, Thermo Scientific NanoDrop 2000). DNA concentration was tweaked to 50 ng/μL by adding nuclease-free water. Finally, the isolated DNA samples were stored at −20 °C in microcentrifuge tubes until further use.^48^

### Genotyping

Genotyping was performed on genomic DNA from tail biopsies by PCR before proceeding with experiments.^49^ For PCR amplification, GoTaq green master-mix (10 μL, Promega, M7123) and 1 μL of each of the forward and reverse primers from 100 μM stock (Integrated DNA Technologies, USA) pre-diluted to 10 μM were added in each 0.2 mL PCR tube. Finally, 5 μL of previously isolated genomic DNA sample and 2 or 3 μL of nuclease-free water were added to each tube to obtain a final reaction volume of 20 μL. PCR was performed using a standard thermocycler (SimpliAmp™ Thermal Cycler). The amplification conditions consisted of an initial denaturation (95 °C for 3 min), followed by 34 cycles of denaturation (94 °C for 30 s), annealing (56–59 °C for 30 s), and extension (72 °C for 30 s) and a final extension at 72 °C for 4 min. PCR products were removed from the thermocycler and maintained at room temperature for a few minutes and then were analyzed by 1.5% (*w*/*v*) agarose gel electrophoresis with ethidium bromide.^40^

For cPLA2, PCR genotyping required three primers, one reverse primer (cPLA 604, 5′-GACTCATACAGTGCCTTCATCAC-3′) and two forward primers (cPLA 3F, 5′-TGTGTACAATCTTTGTGTTGTTTCA-3′ and PgkNeo, 5′-GGGAACTTCCTGACTAGGGG-3′). The PgkNeo and cPLA 604 primers amplify a 300-bp fragment from the knockout allele whereas cPLA 3F and cPLA 604 amplify a 100-bp fragment from the wild-type cPLA2 gene.^50^ For the TNFα-tg genotype, one reverse primer (5′- CGGGCCGATTGATCTCAGC-3′) and one forward primer (5′-GAGGCCAAGCCCTGGTATG-3′) were used. The presence of TNF transgene was validated by the presence of 91-bp products. Lastly, for NF-κB Luc mice, one reverse primer (5′-AGGGTTGGTACTAGCAACGC-3′) and one forward primer (5′-TGGCAGAAGCTATGAAACGA-3′) were used. The presence of the luciferase reporter gene was evidenced via an 182-bp product.^42^

### Preparation of agarose gels and electrophoresis of PCR products

Agarose gels (1.5%) were prepared using agarose of 95% purity (Sigma-Aldrich, A5093) dissolved in 1X Tris-Acetate EDTA (TAE) buffer (40 mM Tris-acetate, 1 mM EDTA, pH 8.3, Fisher Bioreagents BP13324) by heating the solution in an on a hot plate or microwave oven for 3–4 min. The agarose solution was kept at room temperature for about 5 min and then 5 μL of ethidium bromide (10 mg/mL, E7637, Sigma Aldrich) was added to the melted agarose and was immediately poured on a transparent gel casting tray (Bio-Rad PowerPac Basic w/ Wide Mini Sub Cell GT Electrophoresis Unit) fitted with a 10 well comb. The gel tray was placed on the electrophoresis system and the electrophoresis chamber was filled with 1X TAE buffer till about 1.5 cm above the gel. 5–8 μL of each PCR product was loaded into each well and electrophoresis was performed at 6.5 V/cm for 80 min. The power pack supply (1000/500 power supply, Bio-Rad) was set to 90 V. A DNA size marker of 100 bp (Gene ruler 1 kB plus, DirectLoad™ Plus 1 kb DNA Ladder) was used. After the run was complete, the gel images were acquired using a gel documentation system (Biorad Gel Doc XR Imaging System).

### Cell culture experiments and treatments

All cells, *viz*., bone marrow cells, bone marrow-derived macrophages (BMDMs), and RAW264.7 cells (obtained from ATCC) were maintained in Dulbecco's modified Eagle's medium (without pyruvate, Gibco™ 11995073) supplemented with 10% fetal bovine serum (Gibco™ 000044) and 1% penicillin-streptomycin (Gibco™ 15140122) at 37 °C under 5% CO_2_ in a humidified incubator. Cells were detached using 0.05% trypsin (Gibco™ 25300054) if sub-culturing was required. The differentiation media were supplemented with different pyruvate (sigma) concentrations, *viz*., 0.02, 0.06, 0.1, 0.2, 2, 4, 8, and 10 mM for screening. After the screening, low (2 mM) and/or high (4 mM) concentrations of pyruvate were used unless stated otherwise. Mentioning only pyruvate parallels the use of high concentration. In all the experiments, cells were incubated with or without low and high concentrations of pyruvate for 24 h if the sample was to be collected for real-time PCR or 48 h for enzyme-linked immunosorbent assay (ELISA). In the experiments that were subjected to TNFα stimulation, the concentration used for TNFα was 10 ng/mL.

### *In vitro* differentiation of primary BMDMs

Bone marrow cells were isolated from C57BL/6 and TNFα-tg mice. After mice were sacrificed by cervical dislocation, the tibia and femur were isolated under aseptic conditions and washed in PBS containing antibiotic/antimycotic solution (1%, Invitrogen, USA). Both ends of the bones were cut to open the bone medullary cavity and were placed in a 1.5 mL sterile microtube, bone marrow cells were collected by centrifugation at 13,000 *g* for 90 s and isolated cells were suspended in Dulbecco's modified Eagle's medium (Gibco, USA) supplemented with 10% fetal bovine serum (Gibco, USA) and antibiotic/antimycotic (Gibco-Brl #15240-062) with M-CSF (10 ng/mL, 576406, Biolegend) and incubated at 37 °C and 5% CO_2_ for 6 days to allow differentiation.

### RNA extraction and quantitative real-time PCR

Total RNA was extracted from the desired cell or tissue type (BMDMs or colon tissue) using RNeasy Mini Kit (74106, Qiagen). First-strand cDNA synthesis was performed with 1 μg RNA using a High-Capacity cDNA Reverse Transcription Kit (4368813, Applied Biosystems). SYBR green-based (4367659, Applied Biosystems) quantitative PCR was performed in triplicate on the Real-Time PCR System (Applied Biosystems) using specific primers summarized in Table S4. The relative transcription levels of the mRNAs were calculated according to the 2^−ΔΔCt^ formula and reported as a relative mRNA fold change. For the colonic sample, we harvested a piece of approximately 1.0 cm of distal colon in length consistently from the same segment in all the mice. Colonic mucosa was carefully isolated by gently scraping followed by the same RNA isolation procedure.^5^

### Western blot assay

For the mouse samples, the tissues were dissected and snap-frozen in dry ice. Before the experiment, the frozen tissues were homogenized with lysed following the kits' instructions. After protein lysates were prepared, samples were separated by SDS-PAGE and were transferred to a nitrocellulose membrane (162-0115, BIO-RAD) electrophoretically using a wet transfer system. The membrane was blocked using 5% (*w*/*v*) non-fat milk in tris buffered saline Tween solution at room temperature for half an hour, followed by incubation first with an antibody (1:1000 dilution) at 4 °C overnight and a secondary antibody (1:10,000 dilution) at room temperature for 1 h. The immunogenic bands on the membrane were developed by chemiluminescent substrate (Amersham Biosciences, Pittsburgh, PA) and visualized by the GelDoc system. All the antibodies used were from Cell Signaling Technology unless stated otherwise.

### Enzyme-linked immunosorbent assay

The ELISA-based assay was performed to detect the beneficial effects of pyruvate. Cell culture supernatants were collected after 48 h of treatment (treated with or without pyruvate or 5-ASA). Cytokine levels of interleukin (IL)-1β (88-7013, Invitrogen) and IL-6 (88706476, Invitrogen) in cell culture supernatants, sera, or colonic tissues from murine models were detected by sandwich ELISA according to product specifications in ELISA kit. For serum samples, whole blood collected from each mouse was subjected to centrifugation at 3000 rpm for 10 min. For the isolation of protein from tissue lysates, the Abcam Protein Extraction Kit (ab270054) was used following the protocol booklet instructions.

### RNA sequencing and pathway analysis

For bulk RNA sequencing analyses, *in vitro* differentiated BMDMs from TNFα-tg mice were treated with a low or high concentration of pyruvate for 24 h, followed by the extraction of total RNA. RNA sequencing differential expression analysis was performed for one lane of a single-read 50 Illumina HiSeq 4000 run. Per-read per-sample FASTQ files were generated using the bcl2fastq2 Conversion software (v2.20) to convert per-cycle BCL base call files outputted by the sequencing instrument (RTA v2.7.7) into the FASTQ format. The alignment program, STAR (v2.6.1d), was used for mapping reads of 6 samples to the mouse reference genome mm10, and the application Fastq Screen (v.0.13.0) was utilized to check for contaminants. The software, feature Counts (Subread package v1.6.3), was used to generate matrices of reading counts for annotated genomic features. For differential gene, statistical comparisons between groups of samples contrasted by low-treated (2 mM pyruvate), high-treated (4 mM pyruvate), and untreated (TNFα-tg) conditions were performed, the DESeq2 package (R v3.6.1) in the R statistical programming environment was utilized. The sequencing library was in accordance with the manufacturer's instruction by the Illumina TrueSeq mRNA sample preparation kit. Sequencing was conducted on Illumina HiSeq 3000. Genes with adjusted *p*-value ≤0.05 and log2 Fold Change (log_2_FC) of more than 1.5 were considered statistically significant. Panther and KEGG pathways were performed for pathway analysis.

### Nuclear translocation and immunofluorescence staining

BMDMs were cultured on coverslips in 12-well culture plates in the absence or presence of pyruvate (4 mM) and were stimulated by TNFα (10 ng/mL) followed by 6 h of incubation. Immunofluorescence was performed to visualize the subcellular localization of p65. After incubation, the cells were fixed with 4% paraformaldehyde solution at room temperature for 10 min, and then permeabilized in 0.1% Triton X-100 for 10 min, followed by blocked in 1% BSA at room temperature for 1 h. Cells were then incubated with p65 primary antibody (764s, cell Signaling) with dilution 1:50–1:100 at 4 °C overnight. The next day, cells were washed three times in PBS for 10 min by gentle aspiration, and then incubated with anti-Rabbit IgG conjugated with Alexa Fluor® 488 (1:200, A-11008, Invitrogen) at room temperature for 1 h. 4′,6-diamidino-2-phenylindole (DAPI, 1:1000, D1306, ThermoFisher Scientific) was used to stain the nucleus at room temperature for 10 min. The coverslips were removed and mounted on microscope slides with Kaiser's glycerol gelatin and further analyzed and imaged using Leica TCS SP5 confocal system while fluorescence intensity was quantified using ImageJ software.^51^

### Drug affinity responsive target stability (DARTS) assay and mass spectrometry

DARTS was performed according to previously reported methods.^52^ Briefly, RAW264.7 cells were lysed using M-PER™ Mammalian Protein Extraction Reagent (78501, Thermofisher), followed by centrifugation at 18,000 *g* and 4 °C for 10 min. The supernatant (cell lysate) was then incubated with 4 mM pyruvate or PBS (Thermofisher, Cat. 70011044) at room temperature for 1 h and was held on a rotator. This mixture was subjected to digestion by pronase (Sigma, Cat. P5147) at room temperature for 15 min. Samples were boiled after adding 5 × SDS loading dye for SDS-PAGE or Western blot detection. A band with a molecular weight of ∼80 kDa was found to be protected by pyruvate treatment. This band was excised and then analyzed by mass spectrometry, performed at NYU Proteomics Laboratory. All MS/MS spectra were collected using the following instrument parameters: the resolution of 15,000, AGC target of 1e5, maximum ion time of 120 ms, one microscan, 2 *m*/*z* isolation window, fixed first mass of 150 *m*/*z*, and NCE of 27. MS/MS spectra were searched against a Uniprot Human database using Sequest within Proteome Discoverer.

### Cellular thermal shift assay (CETSA)

RAW264.7 cells were treated with pyruvate at 37 °C and 5% CO_2_ for 1 h. Cells were harvested and were equally divided into multiple sterile Eppendorf tubes. Aliquoted cell suspensions were subjected to thermal digestion using a range of temperatures (40, 43, 46, 49, 52, 55, 58, 61, and 64 °C) for 3 min. Samples were subsequently lysed through 3 repetitive freeze–thaw cycles and finally centrifuged at 20,000 *g*. The supernatants were collected for Western blot analysis. After determining the overall melting behavior of the target protein, the isothermal dose response was analyzed. For isothermal dose response analysis, a series of concentrations (ranging from 0.2 to 2000 μM) of pyruvate was used for the treatment of cells, and a constant temperature (55 °C) was selected based on CETSA for thermal denaturation. The remaining steps were followed as previously reported.^53^

### Statistical analyses

Each data was expressed as mean ± standard error of the mean and *p*-values were calculated using a two-tailed student's *t*-test for pairwise comparison of variables, one-way ANOVA for multiple comparisons of variables, and two-way ANOVA for multiple comparisons involving two independent variables. All the experiments were performed at least three times, and all indicated replicates were biological replicates. A *p*-value < 0.05 was considered statistically significant. The numbers of mice employed per genotype were indicated in figure legends. Statistical analysis was carried out using GraphPad Prism ver. 9.4.1 software.

## Results

### Pyruvate attenuated TNFα signaling and inflammation both *in vitro* and *in vivo*

**Figure 1:**
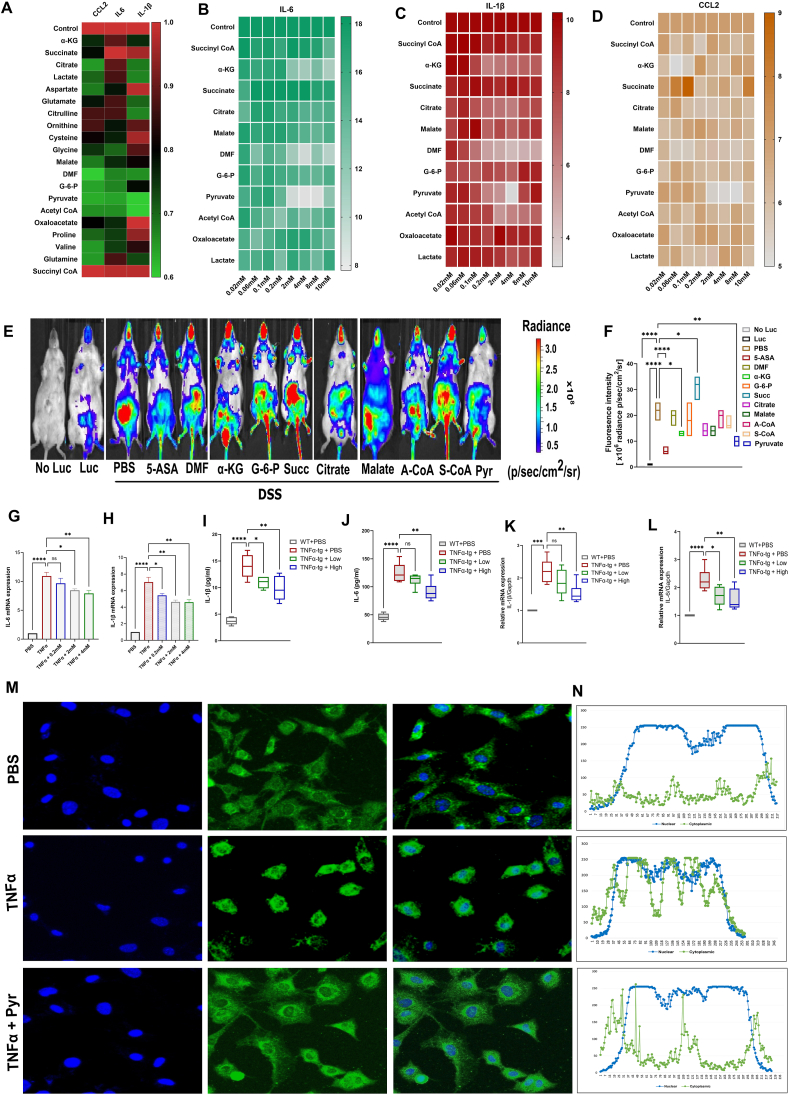
Pyruvate inhibits TNFα/NF-κB signaling and the expression of downstream pro-inflammatory genes. **(A)** BMDMs isolated from WT mice were treated with TNFα in the absence (control) or presence of different metabolites for 24 h. Total RNA was extracted for quantitative reverse transcription PCR analysis for various inflammatory genes. The expression in the TNFα group, normalized against Gapdh, is regarded as 1. **(B**–**D)** RAW264.7 cells were incubated with TNFα in the absence or presence of various concentrations of metabolites shortlisted from (A), as indicated, for 24 h. The mRNA levels of (B) IL-6, (C) IL-1β, and (D) CCL2 were detected by quantitative real-time PCR (*n* = 6). **(E)** NFκB-Luc mice induced with colitis were used to confirm the *in vivo* anti-inflammatory activity of 9 metabolites isolated from the cell-based preliminary screening. After treatment for 5 days with indicated metabolites, the luciferase reporter signal was detected by an *in vivo* imaging system. Representative bioluminescence images were acquired on the 7th day of the experimental time. All images were captured 10 min post-substrate administration with a 60-s exposure. **(F)** Quantification of panel E, representing light emitted ventrally from the mice post-luciferin injection. Statistical analysis was done to compare the treatment groups and control groups (*n* = 6 mice for each group). Data are mean ± standard error of the mean; ∗*p* < 0.05, ∗∗*p* < 0.01, ∗∗∗*p* < 0.001. **(G, H)** mRNA expression of IL-1β and IL-6 with increasing concentrations of pyruvate (as indicated). **(I, J)** mRNA expression of IL-1β and IL-6 with low (2 mM) or high (4 mM) concentration of pyruvate. **(K, L)** The concentration of IL-1β (pg/mL) and IL-6 (pg/mL) in the supernatant was measured by ELISA. Statistical analysis was done to compare the treatment groups and vehicle groups (*n* = 3 mice per group). Data are mean ± standard error of the mean; ∗*p* < 0.05, ∗∗*p* < 0.01, ∗∗∗*p* < 0.001. **(M)** BMDMs were cultured with TNFα (10 ng/mL) in the absence or presence of pyruvate for 6 h. Confocal microscopy was performed to visualize the nuclear translocation of p65. 4′,6-diamidino-2-phenylindole (DAPI) was used to stain the nucleus. Scale bar, 100 μm. *n* = 3 mice per group. **(N)** Quantification of (M), illustrating the nuclear translocation in corresponding groups. TNFα, tumor necrosis factor-alpha; NF-κB, nuclear factor-kappa B; BMDMs, bone marrow-derived macrophages; WT, wild type; IL-6, interleukin-6; IL-1β, interleukin-1beta; CCL2, C–C motif chemokine ligand 2.

In this study, we aimed to perform both cell-based (*in vitro*) and animal-based (*in vivo*) screening to compare the anti-TNFα activity profile of various cellular metabolites including the tricarboxylic acid cycle metabolites (Fig. S1A). In the first round of cell-based screening (Fig. 1A), RAW 264.7 cells were stimulated using TNFα, with or without metabolites for 24 h. The quantitative real-time PCR analysis revealed that 11 out of 20 metabolites substantially down-regulated the expression of inflammatory markers, *viz*., CCL2 (C–C motif chemokine ligand 2), IL-β, and IL-6. These 11 metabolites were further screened with a range of concentrations (0.02 mM–10 mM) on TNFα (10 ng/mL) stimulated RAW264.7 cells (Fig. 1B–D). After three independent implementations for each inflammatory cytokine, eight metabolites showed considerable inhibition of inflammatory activity including α-ketoglutarate, dimethyl fumarate, and pyruvate. Among them, pyruvate showed the best inhibition *in vitro*. Consequently, these eight metabolites plus succinate, which showed pro-inflammatory activity (serving as a negative control) were subjected to a second round of *in vivo* screening (Fig. 1E). Since NF-κB is a well-recognized contributor to the pathogenic mechanism of various gastrointestinal diseases including IBD, we used bioluminescence imaging of NFκB-Luc mice. The NFκB-Luc mice challenged with UC (3 % DSS in drinking water), were employed to confirm the *in vivo* anti-inflammatory activity of nine metabolites isolated from the cell-based preliminary screening, where 5-ASA was used as a positive control (Fig. S1B). After 5 days of co-administration of DSS and indicated metabolites, the luciferase reporter signal was detected by the *in vivo* imaging system. Representative bioluminescence images were acquired on the 7th day of the experimental time and 10 min post-substrate administration with a 60-s exposure. The analysis of fluorescence intensity revealed that pyruvate showed the best anti-inflammatory activity *in vivo*.

Next, we confirmed the anti-TNFα activity of pyruvate in RAW 264.7 cells with three concentrations (0.2 mM, 2 mM, and 4 mM) that showed the best results in preliminary cell-based screening, and it reproduced a concentration-dependent inhibition of inflammatory markers induced by TNFα (Fig. 1G, H). To further authenticate this activity in a different cell type, we isolated bone marrow cells from TNFα-tg. These bone marrow cells were differentiated into BMDMs and subjected to a low (2 mM) and high concentration (4 mM) of pyruvate treatment. Both ELISA (Fig. 1I, J) and quantitative real-time PCR (Fig. 1K, L) demonstrated that pyruvate dose-dependently ameliorated TNFα-induced release and mRNA expression of IL-1β and IL-6, respectively.

Subsequently, we performed RNA sequence analysis for BMDMs isolated from TNFα-tg mice, treated with or without a low and high concentration of pyruvate. This analysis identified over 50,000 differentially expressed genes (Fig. S1C). Out of these, genes with ≥2-fold expression were shortlisted (Fig. S1D). Panther pathway (Fig. S1E) and gene ontology pathway (Fig. S1F) analyses both consistently revealed that pyruvate negatively regulated the inflammatory pathway/response. The heatmap analysis showed a plethora of pro-inflammatory genes to be down-regulated by pyruvate including TNFα-activated NF-κB signaling and its downstream genes (Fig. S1G). The volcano map also demonstrated that IL-27, IL-β, and IL-6 were among the most significantly down-regulated genes (Fig. S1H). Since so far, we evaluated the TNFα-activated NF-κB signaling and its downstream genes, it was rational to verify the effect of pyruvate on TNFα-induced NF-κB nuclear translocation (Fig. 1M, N). It was apparent that with TNFα stimulation, NF-κB was translocated to the nucleus which is normally sequestered in the cytoplasm. However, pyruvate treatment impeded this translocation and hence modulated the transcription of downstream proinflammatory genes. Collectively, the identification and robust validation of pyruvate as a previously unappreciated inhibitor of the TNFα/NF-κB pathway prompted us to determine its potential therapeutic role in DSS-induced UC, a well-accepted TNFα/NF-κB associated inflammatory disease.

### Pyruvate attenuated the clinical disease activity index of DSS-induced colitis

**Figure 2:**
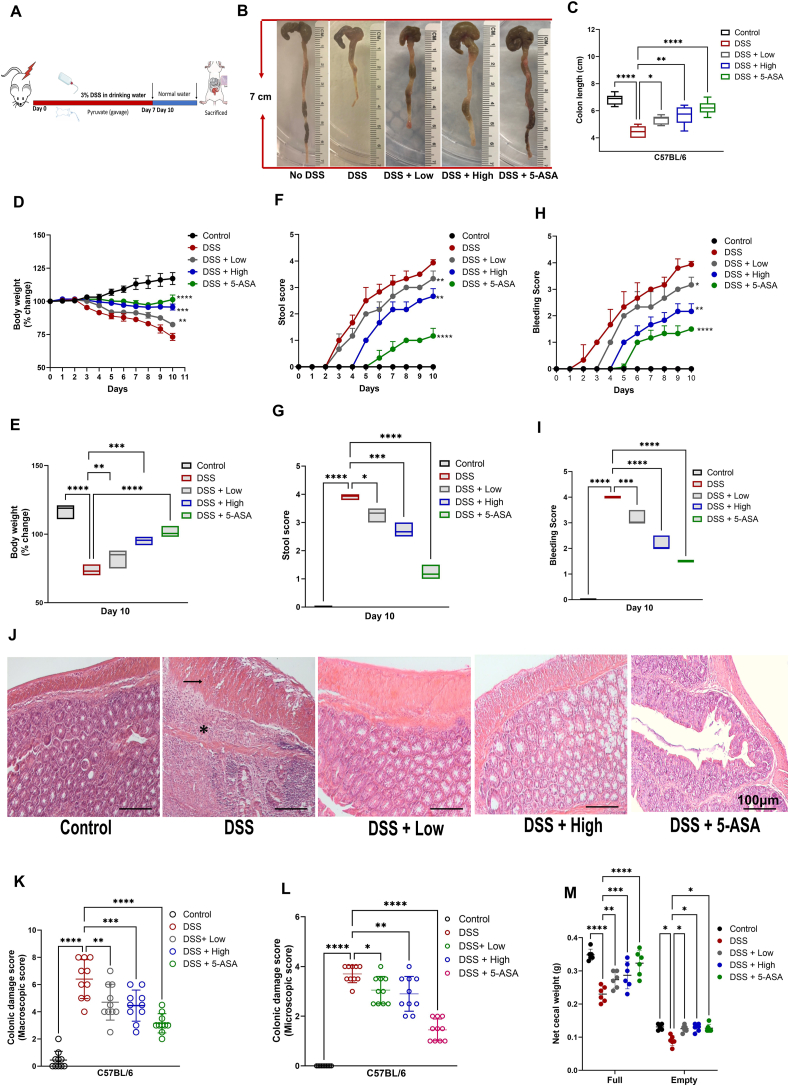
Pyruvate ameliorates DSS-induced colitis. **(A)** The schematic diagram illustrating the major steps of the experimental procedure in the DSS-induced colitis model. The model was established in 8-week-old C57BL/6 mice with *ad libitum* access to drinking water containing 3% DSS for 7 days. Low (40 mg/kg) or high (100 mg/kg) doses of pyruvate and 5-ASA (50 mg/kg, serving as a positive control) were delivered orally from day 0 until the mice were sacrificed. **(B)** Typical gross morphology of the colon on the 10th day from each group showing length differences. **(C)** Colon length in every experimental group. **(D)** Body weight changes (percentage of the original body weight) of different experimental groups. **(E)** Body weight changes on the 10th day of the experiment in different experimental groups. **(F)** Stool score of controls and treated groups. **(G)** Stool score on the 10th day of the experiment in different experimental groups. **(H)** Bleeding score of controls and treated groups. **(I)** Bleeding score of controls and treated groups on the 10th day. **(J)** Hematoxylin and eosin staining of colons from different experimental groups. **(K)** Macroscopic histopathological scoring of the colon. **(L)** Microscopic histopathological scoring of the colon. **(M)** Cecum weight (g) in every experimental group. Statistical analysis was done to compare the treatment groups and vehicle groups (*n* = 6). Data are mean ± standard deviation of the mean; ∗*p* < 0.05, ∗∗*p* < 0.01, ∗∗∗*p* < 0.001. Scale bar, 100 μm. DSS, dextran sodium sulfate.

Experimental colitis was successfully induced as evidenced by its hallmarks, *viz*., bodyweight loss, diarrhea, rectal bleeding, and colon length shortening. Additionally, other illness symptoms including hunched back, raised fur, and decreased mobility because of diarrhea and anemia were also obvious. A schematic diagram of the experimental design is shown in Figure 2A. Our results revealed that oral administration of pyruvate (high and low doses) significantly rescued the shortening of colon length as compared with the DSS group in a dose-dependent manner (Fig. 2B, C). The body weight of mice in the DSS group continuously declined throughout the experimental duration; however, the control group showed an opposite trend. Both the pyruvate treatment groups showed inhibition of body weight loss, and the effect of a high dose of pyruvate was comparable to that of positive drug control 5-ASA (Fig. 2D–G). Both stool and rectal bleeding scores markedly increased in the DSS group which were significantly attenuated in the treatment groups (Fig. 2E, F; Fig. 2H, I and Table S1). The colon damage involving obliteration of histological structure as well as the macroscopic and microscopic scores including focal hyperemia, colonic mucosal ulcerations, and transmural inflammation identified by inflammatory cell infiltration were observed in the DSS group. However, pyruvate administration ameliorated these pathologic changes including the restoration of the local structure of the colon tissue, where the high dose showed a more pronounced effect which was comparable to the 5-ASA group (Fig. 2J–N and Tables S2 and 3). The net cecal weight (both full and empty) was also significantly increased in the treatment groups as compared with the DSS group (Fig. 2M).

### Pyruvate attenuated colonic proinflammatory cytokines and preserved the tight junction function in the intestinal epithelium

**Figure 3:**
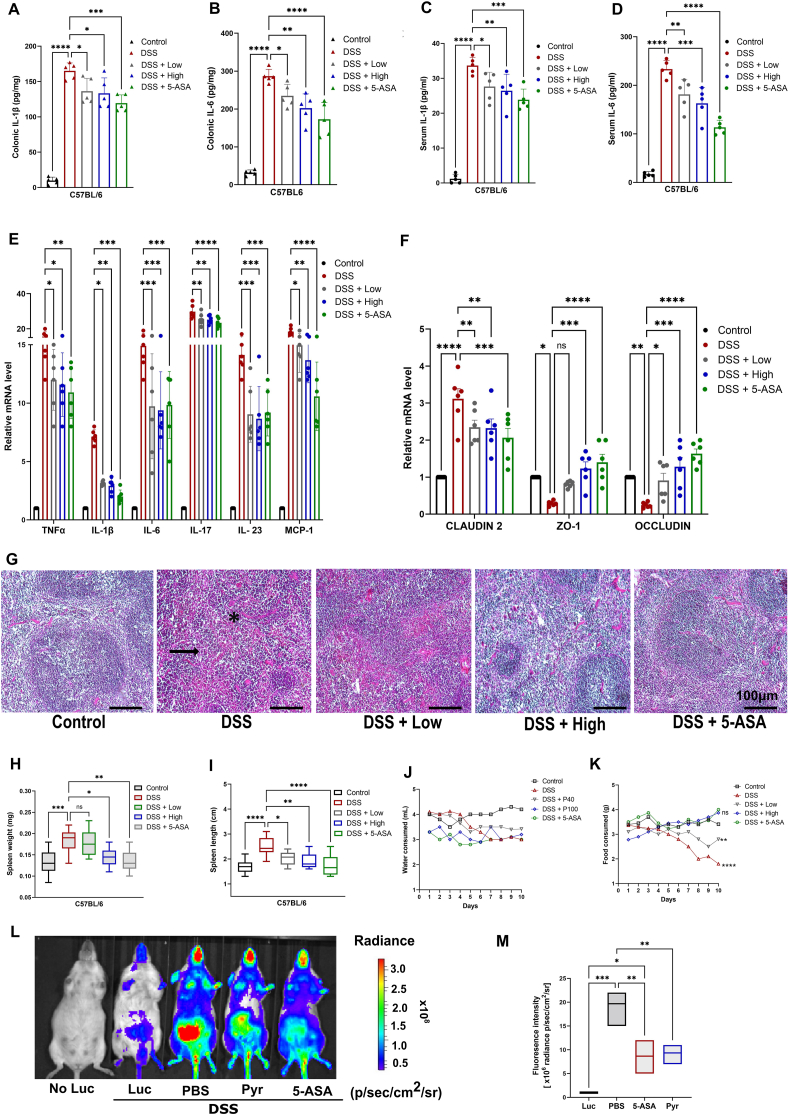
Pyruvate reduces the level of pro-inflammatory cytokines and decreases intestinal permeability in DSS-induced colitis. **(A, B)** IL-1β (A) and IL-6 (B) in colon tissues, assayed by ELISA. **(C, D)** Serum levels of IL-1β and IL6 in different experimental groups were measured by ELISA. **(E)** Gene expression data of Th1 cytokine (TNFα, IL-1β), Th2 cytokines (IL-6), Th17 cytokine (IL-17, IL-23), and chemokine (MCP-1) in colonic tissue from different experimental groups, assayed by quantitative real-time PCR. **(F)** mRNA expression of colonic mucosal levels of tight junction proteins in different treatment groups, measured by quantitative real-time PCR. **(G)** Hematoxylin and eosin staining of spleen tissues. The black arrow indicates damage to the white pulp. **(H)** Weight of the spleen (mg) in different experimental groups. **(I)** Spleen length in different groups. **(J)** Water consumption (3% DSS in drinking water with or without other treatment). **(K)** Food intake during the experimental period. **(L)***In vivo* imaging system of NFκB-Luc reporter mice validating pyruvate treatment in attenuating DSS-induced colitis. Representative bioluminescence images were acquired on the 7th day of the experimental time. All images were captured 10 min post-substrate administration with a 60-s exposure. **(M)** Quantification of (L), representing light emitted ventrally from the mice post-luciferin injection. Statistical analysis was done to compare the treatment groups and vehicle groups (*n* = 6). Data are mean ± standard error of the mean; ∗*p* < 0.05, ∗∗*p* < 0.01, ∗∗∗*p* < 0.001. Scale bar, 100 μm. DSS, dextran sodium sulfate; IL-6/17/23, interleukin-6/17/23; IL-1β, interleukin-1beta; TNFα, tumor necrosis factor-alpha; MCP-1, monocyte chemoattractant protein-1.

Since we found that pyruvate was an inhibitor of TNFα/NF-κB signaling, we next assessed the colonic as well as the serum level of proinflammatory cytokines. Compared with the control group, the levels of IL-1β and IL-6 were drastically increased in the DSS group in both colonic tissue (Fig. 3A, B) and sera (Fig. 3C, D). However, the treatment groups showed significant overall attenuation in their levels. We also investigated the relative mRNA expression of Th1 cytokines (TNFα and IL-1β), Th2 cytokine (IL-6), Th17 cytokines (IL-6 and IL-23), and chemokine (monocyte chemoattractant protein-1/MCP-1) in the colonic tissue. All the above cytokines were increased following DSS stimulation. However, pyruvate treatment attenuated this trend, with significance achieved for all pro-inflammatory cytokines (Fig. 3E).

We further evaluated intestinal barrier dysfunction as it is a considerable feature in the pathogenesis of clinical as well as experimental colitis. Since the intestinal barrier function is affected by tight junction function, we, therefore, analyzed the mRNA expression of three characteristic tight junction proteins: claudin-2, zonula occludens-1 (ZO-1), and occludin. The pore-forming protein (claudin-2) was elevated after DSS stimulation as compared with the control group, corresponding to intestinal barrier damage. However, this trend was effectively alleviated by pyruvate (Fig. 3F). Furthermore, the expression of ZO-1 and occludin were decreased significantly in the DSS group, yet again indicating a compromised tight junction function, where a high dose of pyruvate markedly rescued this decrease. The low dose, however, was irresponsive toward the expression level of ZO-1 but significantly elevated the expression of occludin (Fig. 3F).

As experimental colitis progresses, another feature that evaluates immune cell infiltration is the gross and histological characteristics of the spleen. Hence, we further analyzed the degree of splenic injury by H&E staining. Our results revealed that DSS administration obliterated the spleen structure by causing damage ranging from diffuse sinusoidal edema to displaying red pulp hyperemia. In addition, the presence of necrosis in follicles of white pulp was also observed (Fig. 3G). DSS stimulation also showed an increase in spleen weight and length marking the immune cell infiltration. Nevertheless, a high dose of pyruvate mitigated both histological as well as morphological damage by attaining near-normal histology of splenic tissue in addition to reducing the splenic weight and length (Fig. 3G–I). The low dose of pyruvate was less impactful in recuperating the normal histology and remained ineffective concerning spleen weight. However, it reduced the splenic length significantly (Fig. 3G–I).

We also evaluated the water and food intake as it is associated with disease outcome, in different groups of experimental mice. We observed that the DSS-administered group significantly declined the water and food intake starting from the 4th day. While the treated group did not show any loss of appetite or reduced water intake except for the low-dose group which showed a significant decline in food intake after the 6th day (Fig. 3J, K).

The NFκB-Luc mice induced with colitis were used once again to substantiate the role of pyruvate in mitigating DSS-induced colitis. The analysis showed attenuation of fluorescence intensity by a high dose of pyruvate which was also comparable to 5-ASA, confirming its therapeutic role in murine colitis (Fig. 3L and M).

### cPLA2 is a previously unrecognized target of pyruvate

**Figure 4:**
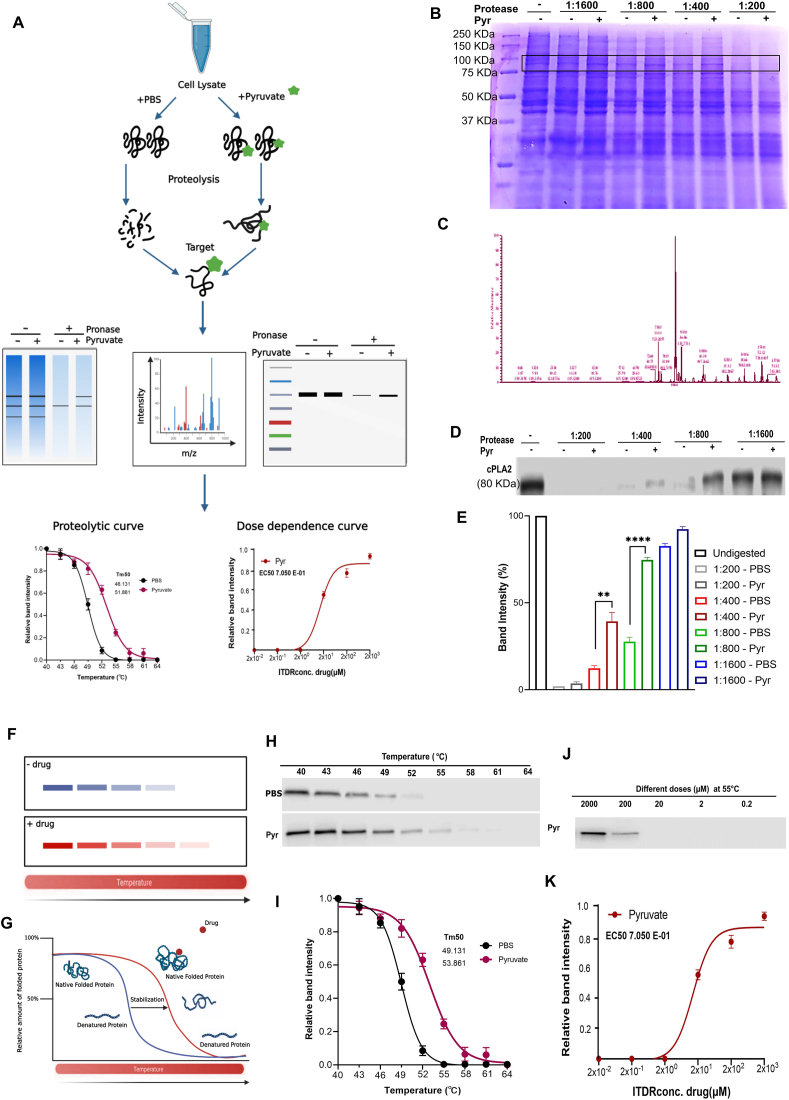
cPLA2 was identified as a novel target of pyruvate. **(A)** Schematic representation of drug affinity responsive target stability (DARTS). **(B)** Coomassie blue staining of DARTS assay gel. The band with a molecular weight of approximately 80 kDa was protected by pyruvate treatment (indicated in a black box). **(C)** cPLA2 adapted image from mass spectrometry. **(D)** DARTS followed by Western blot using an anti-cPLA2 antibody to confirm pyruvate's binding to cPLA2. **(E)** Quantification of (D) showing relative band intensity in with or without pyruvate groups. **(F)** Schematic representation of cellular thermal shift assay (CETSA). **(G)** Schematic representation of CETSA outlining the principle. **(H)** RAW 264.7 cell lysate was denatured under a range of heating temperatures. The protein level of cPLA2 in control and pyruvate-treated groups was assayed using Western blot and densitometry analysis curve. **(I)** The associated curve of CETSA showing a shift in the melting curve with pyruvate treatment. **(J)** Isothermal dose response (ITDR) with a series of pyruvate concentrations at a constant heating temperature of 55 °C. The protein level of cPLA2 was measured using Western blot. **(K)** ITDR associated curve showing relative soluble fraction (*n* = 3). cPLA2, cytosolic phospholipase A2.

To elucidate the molecular mechanism of pyruvate inhibition of inflammation, we focused on identifying its direct binding target(s). For this purpose, DARTS assay was employed with its strategy elaborated in Figure 4A. After proteolytic digestion of cell lysate, the protein mixture was separated by sodium dodecyl sulfate-polyacrylamide gel electrophoresis (SDS-PAGE) and the results indicated that a band with a molecular weight of ∼80 kDa was protected by pyruvate (Fig. 4B). This band was excised and subjected to mass spectrometry for protein identification (Fig. 4C). This led to the identification of *PLA2G4A* encoding cPLA2 as a potential target, which is a critical mediator of inflammation.^54^ To confirm this target, DARTS samples with a series of protease-to-cell lysate ratios were subjected to a Western blot analysis which indicated that the cPLA2 band was protected by pyruvate against enzymatic digestion (Fig. 4D, E).

To further validate the association of pyruvate and cPLA2, a CETSA was performed. CETSA grants quantification of the change in thermal denaturation of a target protein under different conditions, including those of variable temperatures (Fig. 4H, I) and concentrations of the compound of interest (Fig. 4J, K). It was clear that pyruvate prevented denaturation of cPLA2 and kept more cPLA2 in the soluble condition under several temperatures, remarkably noticeable at 49 °C, compared with PBS (Fig. 4H). The melt curve also indicated a substantial shift and change of melting temperature in the presence of pyruvate (melting temperature = 53.8 °C) as compared with the PBS group (melting temperature = 49.1 °C) (Fig. 4I). To confirm the performance of CETSA at 55 °C, we obtained isothermal dose response and analyzed its curve (Fig. 4J, K). This data revealed that pyruvate prevented cPLA2 denaturation in a dose-dependent manner, with an EC50 of 7.050E-01 (Fig. 4K).

### cPLA2 is required for pyruvate to alleviate pathogenesis features in DSS-induced colitis

**Figure 5:**
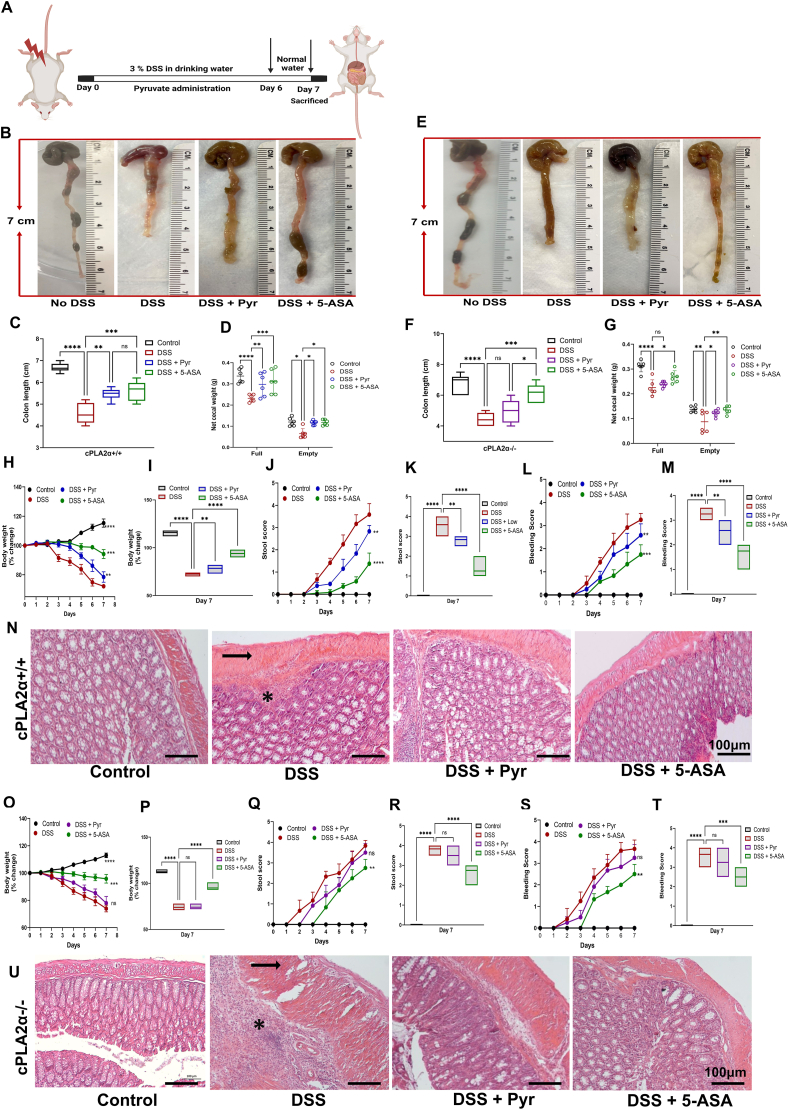
Pyruvate's therapeutic effects against DSS-induced colitis were lost in cPLA2 ablated mice. **(A)** The schematic diagram illustrating the major steps of the experimental procedure in the DSS-induced colitis model in *cPLA2α*^*+/+*^ (WT) and *cPLA2α*^*−/−*^ (cPLA2 KO). **(B**–**D)** Analysis of DSS-induced *cPLA2α*^*+/+*^ mice. (B) Typical gross morphology of colon on the 7th day showing length differences. (C) Colon length in each experimental group. (D) Cecum weight (g) in each experimental group. **(E**–**G)** Analysis of DSS-induced *cPLA2α*^*−/−*^ mice. (E) Typical gross morphology of colon on the 7th day. (F) Colon length in each experimental group. (G) Cecum weight (g) in each experimental group. (**H–N**) Disease activity scoring for *cPLA2α*^*+/+*^ mice. (H) Body weight changes (percentage of the original body weight). (I) Body weight change on the 7th day of the experiment. (J) Stool score of controls and treated groups. (K) Stool score on the 7th day of the experiment. (L) Bleeding score of controls and treated groups. (M) Bleeding score on the 7th day of the experiment. (N) Hematoxylin and eosin staining of colons from different experimental groups of DSS-induced colitis model in *cPLA2α*^*+/+*^ mice. **(O–U)** Disease activity scoring for *cPLA2α*^*−/−*^ mice. (O) Body weight changes (percentage of the original body weight). (P) Body weight change on the 7th day of the experiment. (Q) Stool score of controls and treated groups. (R) Stool score on the 7th day of the experiment. (S) Bleeding score of controls and treated groups. (T) Bleeding score on the 7th day of the experiment. (U) Hematoxylin and eosin staining of colons from different experimental groups of DSS-induced colitis model in *cPLA2α*^*−/−*^ mice. Statistical analysis was done to compare the treatment groups and vehicle groups (*n* = 6). Data are mean ± standard error of the mean; ∗*p* < 0.05, ∗∗*p* < 0.01, ∗∗∗*p* < 0.001. Scale bar, 100 μm. DSS, dextran sodium sulfate; cPLA2, cytosolic phospholipase A2; WT, wild type; KO, knockout.

To confirm the dependence of pyruvate's effects on cPLA2 *in vivo*, we generated cPLA2 KO mice by crossbreeding strategy (Fig. S2A) which was confirmed by genotyping (Fig. S2B). We established DSS-induced colitis in cPLA2 KO mice, and their wild-type controls treated with pyruvate (Fig. 5A). The disease activity scoring revealed that DSS-administered mice showed significant shortening of colon length with reduced cecal weight (Fig. 5B–G), body weight loss (Fig. 5H, I) with elevated scores for stool (Fig. 5J, K), and rectal bleeding (Fig. 5L, M). It was reassuring to see that pyruvate corroborated its protective effects in cPLA2 WT, *i.e.*, reduced bleeding and stool scores, body weight loss, and colon shortening. However, the effects of pyruvate in alleviating the clinical scores were compromised in cPLA2 KO mice (Fig. 5O–T).

Histological analyses also showed that the colonic damage, both macroscopic and microscopic, was rescued by pyruvate administration in WT mice (Fig. 5N; Fig. S3A, B), although cPLA2 deficient mice failed to retain the beneficial effects and abolished the restoration of the normal structure of colon tissue (Fig. 5U; Fig. S3C, D). However, the 5-ASA group was still efficient in reducing the chief clinical signs of colitis regardless of genotype.

The food intake pattern in WT mice demonstrated no loss of appetite in the treatment groups as compared with the DSS-stimulated group, which showed a considerable decline on the 3rd day in WT mice (Fig. S3E). The water intake, however, was non-significant irrespective of the treatment group, except for the DSS-stimulated group on the 7th day (Fig. S3F). However, in KO mice, this loss of appetite remained comparable between the DSS-stimulated group and the pyruvate-administered group as pyruvate failed to recuperate the diseased state in cPLA2 deficiency (Fig. S3G). The water intake remained non-significant throughout the genotypes. The 5-ASA group consistently displayed no notable change in food or water consumption in WT or KO mice. Collectively, these results indicate that the mechanism of pyruvate in mitigating the clinical disease activity index of DSS-induced colitis depends on cPLA2.

### Pyruvate was ineffective in alleviating the DSS-induced host inflammation and in preserving intestinal membrane permeability in cPLA2-deficient mice

**Figure 6:**
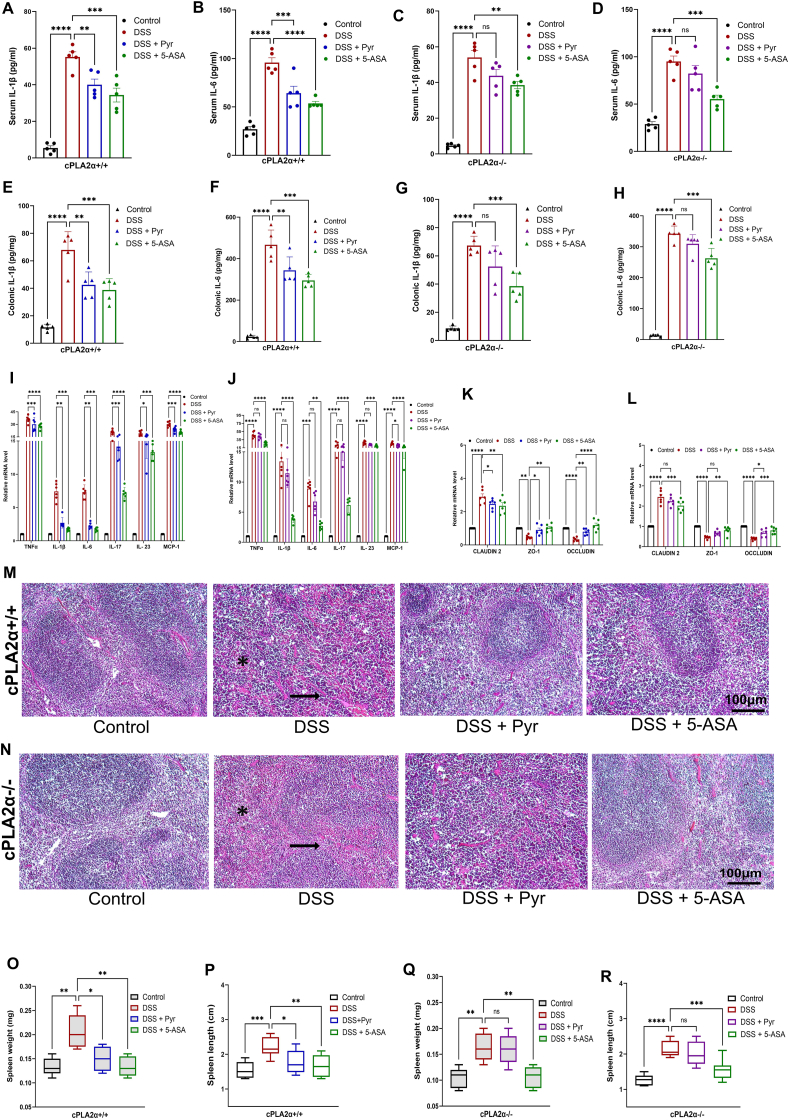
Pyruvate lost its activity in mitigating the levels of pro-inflammatory cytokines and in maintaining the intestinal barrier in *cPLA2α*^*−/−*^ mice. **(A**–**D)** Serum levels of IL-1β and IL-6 in different experimental groups of (A, B) *cPLA2α*^*+/+*^ mice and (C, D) *cPLA2α*^*−/−*^ mice, measured by ELISA. **(E**–**H)** ELISA-based quantification of cytokines IL-6 and IL-1β in colon tissues of (E, F) *cPLA2α*^*+/+*^ mice and (G, H) *cPLA2α*^*−/−*^ mice. **(I, J)** Gene expression data of Th1 cytokines (TNFα, IL-1β), Th2 cytokine (IL-6), Th17 cytokines (IL-17, IL-23), and chemokine (MCP-1) in colonic tissue from (I) *cPLA2α*^*+/+*^ and (J) *cPLA2α*^*−/−*^ mice, assayed by quantitative real-time PCR. **(K, L)** mRNA expression of colonic mucosal levels of tight junction proteins in (K) *cPLA2α*^*+/+*^ mice and (L) *cPLA2α*^*−/−*^ mice, assayed by quantitative real-time PCR. **(M, N)** Hematoxylin and eosin staining of spleen tissues. The black arrow indicates damage of white pulp in (M) *cPLA2α*^*+/+*^ mice and (N) *cPLA2α*^*−/−*^ mice. **(O)** Weight of the spleen (mg) in different experimental groups. **(P)** Spleen length in different groups of *cPLA2α*^*+/+*^ mice. **(Q)** Weight of the spleen (mg) in different experimental groups. **(R)** Spleen length in different groups of *cPLA2α*^*−/−*^ mice. Statistical analysis was done to compare the treatment groups and vehicle groups (*n* = 6). Data are mean ± standard error of the mean; ∗*p* < 0.05, ∗∗*p* < 0.01, ∗∗∗*p* < 0.001. Scale bar, 100 μm. cPLA2, cytosolic phospholipase A2; IL-1β, interleukin-1beta; IL-6/17/23, interleukin-6/17/23; TNFα, tumor necrosis factor-alpha; MCP-1, monocyte chemoattractant protein-1.

The effect of pyruvate was further evaluated on proinflammatory cytokine levels in colitis-induced cPLA2 WT and KO mice. As anticipated, the serum (Fig. 6A, B) and colonic (Fig. 6E, F) levels of IL-1β and IL-6 were reduced after pyruvate and 5-ASA treatment in WT mice as compared with the DSS-challenged group. However, pyruvate could not reduce the levels of IL-1β and IL-6 in serum (Fig. 6C, D) or colonic tissue (Fig. 6G, H) in cPLA2 deficient mice.

The results from the mRNA expression of Th1 cytokines (TNFα and IL-1β), Th2 cytokine (IL-6), Th17 cytokines (IL-17 and IL-23), and chemokine (MCP-1) in the colonic tissue suggested that pyruvate retained its anti-inflammatory activity in WT mice by reducing the expression of the cytokines (Fig. 6I). However, pyruvate was unable to reduce the expression for most of these cytokines except for MCP-1 in KO mice (Fig. 6J).

Next, we evaluated the modulation of gut permeability and epithelial paracellular pathways in colitis-induced WT and KO mice. The results showed that the transcription levels of claudin-2 were significantly down-regulated by pyruvate and 5-ASA treatment, which was otherwise elevated in the WT mice of the DSS group. The other tight junction proteins, *viz*., ZO-1 and occludin were significantly enhanced by pyruvate and 5-ASA, as compared with the DSS group (Fig. 6K). Besides, cPLA2 deficient mice showed that pyruvate's treatment was ineffective with regard to claudin-2 and zo-1 expression, however, it was noteworthy to see that pyruvate was able to down-regulate the expression of occludin (Fig. 6L). On the other hand, 5-ASA consistently showed a similar trend of significantly down-regulating the expression of pro-inflammatory cytokines and modulating the expression of tight junction proteins in both WT and KO mice.

When histopathology was compared between WT and KO mice for splenic damage, it was found that there was severe inflammatory injury and deterioration of splenic tissue in the DSS-challenged group. Pyruvate as well as 5-ASA improved this damage by restoring the near-normal histology of splenic tissue (Fig. 6M). However, where 5-ASA consistently reduced the histopathological damage in the spleen, pyruvate failed to achieve these results in KO mice (Fig. 6N). Also, the weight (Fig. 6O) and length (Fig. 6P) of the spleen in WT mice were reduced by 5-ASA treatment, indicating a resolved inflammatory state. When these results were evaluated in the cPLA2 deficient mice, the spleen weight (Fig. 6Q) and length (Fig. 6R) remained unaffected by pyruvate treatment and were comparable to DSS-administered mice implying its failure to resolve the overall inflammatory state. Taken together, these outcomes indicate that cPLA2 is indispensable for the therapeutic effects of pyruvate in attenuating DSS-induced colitis.

## Discussion

UC is a complex, immune-mediated inflammatory disorder characterized by complex etiology involving epithelial barrier defects, dysregulated mucosal immune responses, dysbiosis, genetic predisposition, and environmental factors.^55^ Currently, a number of drugs are available for the management of clinical symptoms associated with intestinal inflammation, including salicylates, glucocorticoids, and immunosuppressants. These therapies have shown to be efficacious, however, their long-term intake has potential adverse effects that concern patient compliance and quality of life.^7^

Among the immune-regulatory factors, the inflammatory reaction has long been recognized as the key mechanism in the pathophysiology of chronic colitis.^56^ TNFα being at the apex of the inflammatory cascade is the uncontested master regulator of inflammation. It activates NFκB nuclear translocation and signaling consequently up-regulating the transcription of proinflammatory cytokines, including IL-1β and IL-6, which are the key contributors to intestinal mucosal inflammation.^57^ Because TNFα expands the local and systemic inflammation, it is known to trigger a dysregulation in the distribution and structure of tight junction proteins followed by an impaired epithelial paracellular barrier function.^58^^,^^59^ Hence, it is credible to screen for a drug candidate that has anti-TNFα activity, with a safe administration and effective outputs.

Therefore, in this study, we performed binary sets of screenings using a variety of cellular metabolites and isolated pyruvate to show the most potent anti-TNFα action. Pyruvate not only reduced the expression levels of key proinflammatory cytokines, *viz*., IL-1β and IL-6 *in vitro* but also validated the same results when a model of systemic inflammation was used. Pyruvate also significantly reduced the secretion of IL-1β and IL-6 in TNFα-tg mice. Interestingly, pyruvate did not affect the binding of TNFα to its cell surface receptors (data not shown). Another strategy in treating UC is the pharmacological attempts to block NFκB signaling.^18^ NFκB is known to be evidently activated in UC patients because of its proficiency to stimulate the expression of various proinflammatory genes. These cytokines stimulate macrophage migration thus amplifying mucosal inflammation.^56^^,^^60^ Our study presents compelling evidence that pyruvate could prevent this signaling *in vivo* using NFκB luciferase reporter mice. Likewise, TNFα induced NFκB nuclear translocation in the primary BMDMs was also constrained by pyruvate (Fig. 1).

DSS challenge induced significant colon damage and intestinal barrier disruption with inflammatory cell infiltration. These pathological manifestations were comparable to UC patients and hence marked the successful establishment of a murine colitis model. Oral administration of pyruvate decreased the disease activity scores and alleviated the histopathological changes in the colon (Fig. 2).^3^ To evaluate the inflammatory state of the colon, the secretion of proinflammatory cytokines was assessed in colonic tissue as well as in blood serum and we observed that pyruvate highly mitigated the inflammatory state. The expression of Th1, Th2, and Th17 cytokines and chemokine MCP-1 were significantly attenuated in the colonic tissue by pyruvate. Furthermore, several studies have explored the impact of pyruvate on T-cell differentiation and function.^61^ Pyruvate also demonstrated a reduction in the production of pro-inflammatory cytokines such as TNFα, IL-1β, and IL-6 by pyruvate, which are essential for the differentiation of naïve T cells into effector T cell subsets like Th1, Th2, and Th17. Furthermore, pyruvate promotes the proliferation of regulatory T cells, which are anti-inflammatory. Hence, by altering the balance of pro- and anti-inflammatory cytokines, pyruvate creates an environment less favorable for the differentiation of naïve T cells into inflammatory effector T cells.^62^

The intestinal mucosal barrier is the first line of defense in an antagonistic environment, principally formed by the tight junctions of epithelial cells. These tight junctions are a combination of transmembrane proteins (occludins and claudins) and accessory proteins (zonula occludens). As the name indicates, they seal the gaps between the junctions of intestinal epithelial cells firmly and hence retain the membrane integrity by confining the pathogens and harmful antigens in the lumen. For the same reason, the patients with UC present increased membrane permeability due to altered expression of proteins regulating tight junctions which facilitates the antigen access from the lumen exacerbating immune response.^63^ It was encouraging to find that pyruvate administration was indisputably effective in regulating the expression of tight junctions preserving intestinal mucosal barrier function for attenuating UC in mice. Splenomegaly and swelling of the spleen are usually associated with the magnitude of inflammation of the gut mucosa. Thus, while evaluating the degree of spleen damage by H&E staining, we observed that DSS altered the spleen structure (white pulp) with an evident accumulation of inflammatory cells.^64^^,^^65^ However, pyruvate notably restored the spleen histology commemorating a resolved inflammatory response (Fig. 3).

DARTS, a universally applicable method to detect the interaction between small molecules and their target proteins,^66^^,^^67^ together with proteomics, CETSA, and isothermal dose response, identified cPLA2 as a formerly unknown target of pyruvate (Fig. 4). cPLA2 phosphorylation is essential in hydrolyzing membrane phospholipids, leading to arachidonic acid mobilization,^68^, ^69^, ^70^ which subsequently stimulates NFκB nuclear translocation leading to pro-inflammatory cytokine release initiating the inflammation.^71^ We also showed that pyruvate not only targeted cPLA2 but was indispensable for its therapeutic action in DSS-induced colitis (Figure 5, Figure 6).

cPLA2 is a calcium-dependent enzyme that produces arachidonic acid and another fatty acid from phospholipids by hydrolyzing the sn-2 acyl bond position. It is known to play crucial roles in a plethora of inflammatory diseases from autoimmune to neurodegenerative diseases.^72^^,^^73^ Its role in the pathogenesis of multiple sclerosis,^74^ postischemic brain injury,^75^ tumor angiogenesis,^76^ and leukoencephalopathy^77^ is well-documented. Nevertheless, even after diligent endeavors, most of the available inhibitors for cPLA2 showed malabsorption, poor distribution, and toxicity.^78^^,^^79^ Hence, well-tolerated, safe, and cost-effective options are certainly required, and pyruvate just fits the bill. Pyruvate has been used in clinics as an oral dietary supplement for the treatment of hypercholesterolemia proving that systemic administration of pyruvate ensures safety, ease of digestion, and patient compliance.^80^^,^^81^

Overall, our work has laid down a strong foundation for pyruvate to be used as an anti-colitis agent, but a few limitations to our study were also noted. First, we conducted this study using the acute colitis model and do not have data on the role of pyruvate in the prognosis of the chronic model of colitis. Second, as luminal bacteria are known to play a crucial role in the development of colitis, we did not assess the role of pyruvate in dysbiosis. Third, female mice were not included and thus no sex-dependent study was performed. Lastly, we only used the global cPLA2 knockout, however, the use of the macrophage-specific ablation of cPLA2 would have been a more powerful approach. Having pointed out these issues, we plan to address them in our subsequent study.

In conclusion, we demonstrated that oral administration of pyruvate dramatically attenuated the pathological manifestations and preserved the permeability of the epithelial barrier in DSS-induced colitis. This study unraveled cPLA2 as a previously unrecognized target of pyruvate, making it the first research that delivers mechanistic insight into the comprehension of pyruvate as anti-colitic potential. Therefore, the promising amalgamation of safe oral administration with anti-inflammatory properties makes us envisage pyruvate as a viable therapeutic option for the treatment of UC, and probably other TNFα/NFκB associated diseases and conditions as well.

## CRediT authorship contribution statement

**Sadaf Hasan:** Writing – review & editing, Writing – original draft, Visualization, Validation, Software, Project administration, Methodology, Investigation, Formal analysis, Data curation, Conceptualization. **Nabil Ghani:** Writing – review & editing, Writing – original draft. **Xiangli Zhao:** Writing – review & editing, Writing – original draft, Validation. **Julia Good:** Validation, Methodology, Data curation. **Chuan-ju Liu:** Writing – review & editing, Visualization, Validation, Supervision, Resources, Funding acquisition, Formal analysis, Conceptualization.

## Funding

This work was partially supported by the US National Institutes of Health (NIH) research grants R01AR062207, R01AR061484, R01AR076900, R01AR078035, and R01NS070328.

## Conflict of interests

The authors have no conflict of interests to declare.
