# Supplementary material for: Exogenous pyruvate is therapeutic against colitis by targeting cytosolic phospholipase A2
Source: Genes Dis. 2025 Feb 22;12(5):101571. doi: 10.1016/j.gendis.2025.101571 (PMC12221594; doi:10.1016/j.gendis.2025.101571)
Supplement: Multimedia component 1 [file mmc1.docx]

**
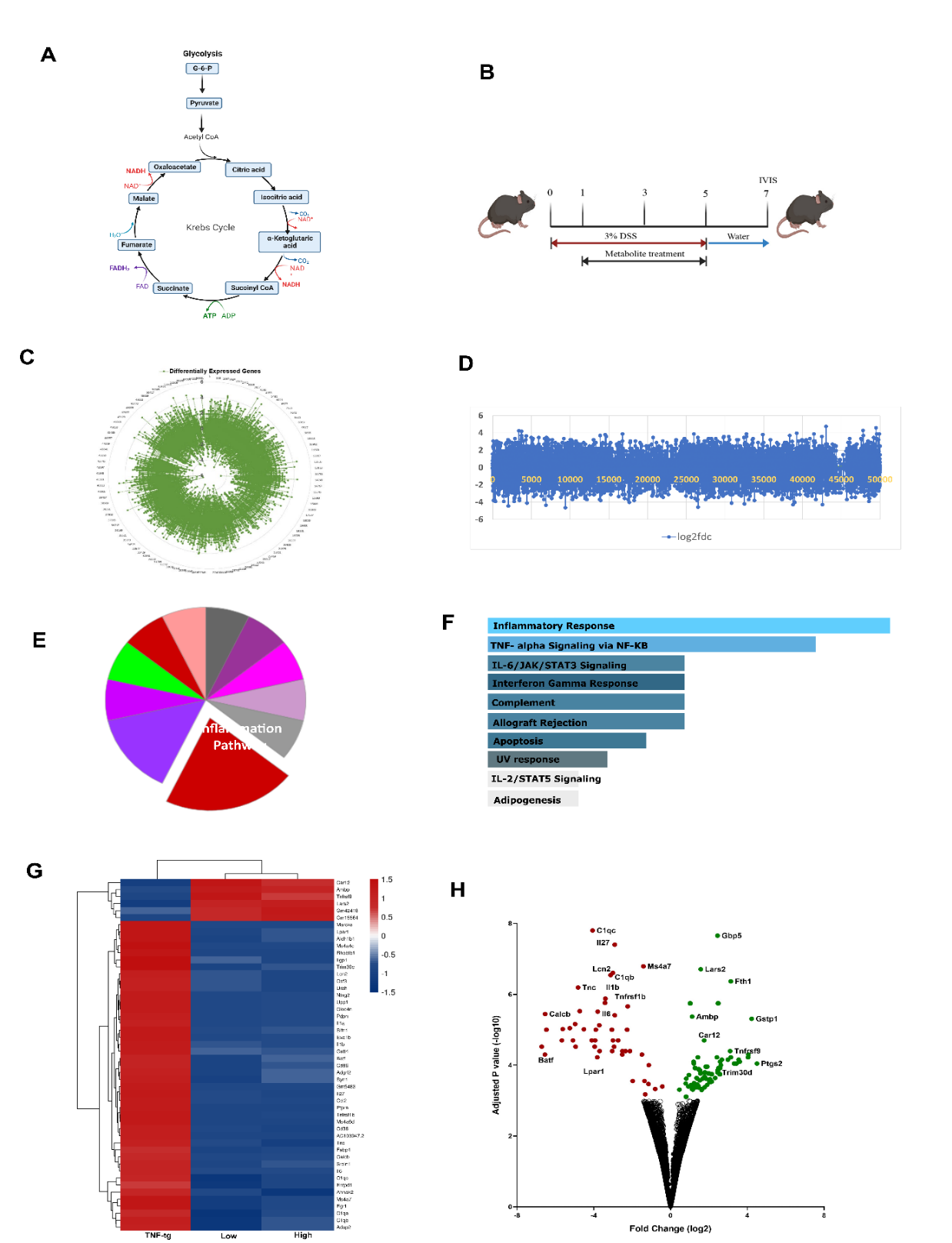
**

**Figure S1 (A)** Schematic representation highlighting the cross-section of glycolysis and TCA cycle and the metabolites used in IVIS screening. **(B)** Diagrammatic representation of the method adopted for IVIS. **(C-H)** Pyruvate attenuated inflammatory pathway as revealed by Pathway analyses using RNA-Seq data of *in vitro* differentiated BMDMs from TNFα-tg mice. *In vitro* differentiated BMDMs (TNFα-tg mice) were treated in the absence or presence of Low (2 mM) and High (4 mM) concentrations of Pyruvate for 24 hours. Total RNA was extracted for RNA-seq. **(C)** Radar plot showing > 50,000 differentially regulated genes (DEGs). **(D)** Scatter plot showing the sorted data of upregulated and downregulated genes. **(E)** Panther pathway **(F)**. KEGG pathway, analyses revealed that the inflammatory pathway was significantly suppressed by pyruvate. **(G)** Heatmap showing significantly upregulated and downregulated genes including classic TNFα inducible genes. **(H)** Volcano plot of differentially expressed genes in the treatment group (High) vs control (TNFα-tg). Each dot represents one gene, the x-axis represents the log 2-fold change (FC), and the y-axis represents -log10 (p values). (n=6).

**
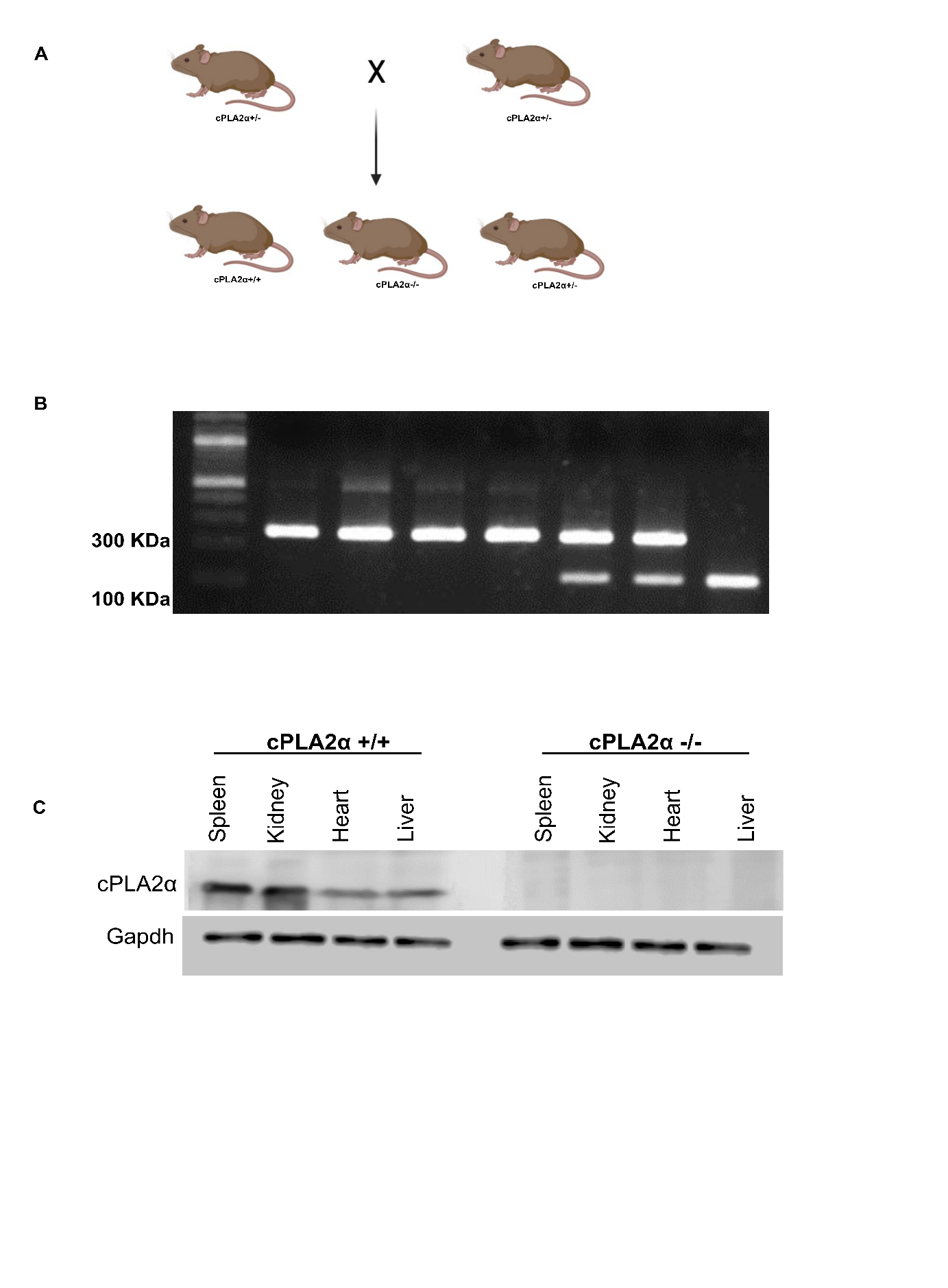
**

**Figure S2** (**A**) Diagrammatic representation breeding through cPLA2 heterozygous crosses to obtain homozygous wildtype (cPLA2α +/+) and homozygous knockout (cPLA2α-/-). (**B**) Genotyping confirming the 100 KDa band for the homozygous wildtype band (cPLA2α +/+), 300 kDa band for the homozygous knockout band (cPLA2α -/-) and the presence of both bands corresponds to a heterozygous strain (cPLA2α -/+). (**C**) Western blot using lysates from different organs of cPLA2α +/+ and cPLA2α -/- mice to ensure global ablation of the cPLA2 gene.


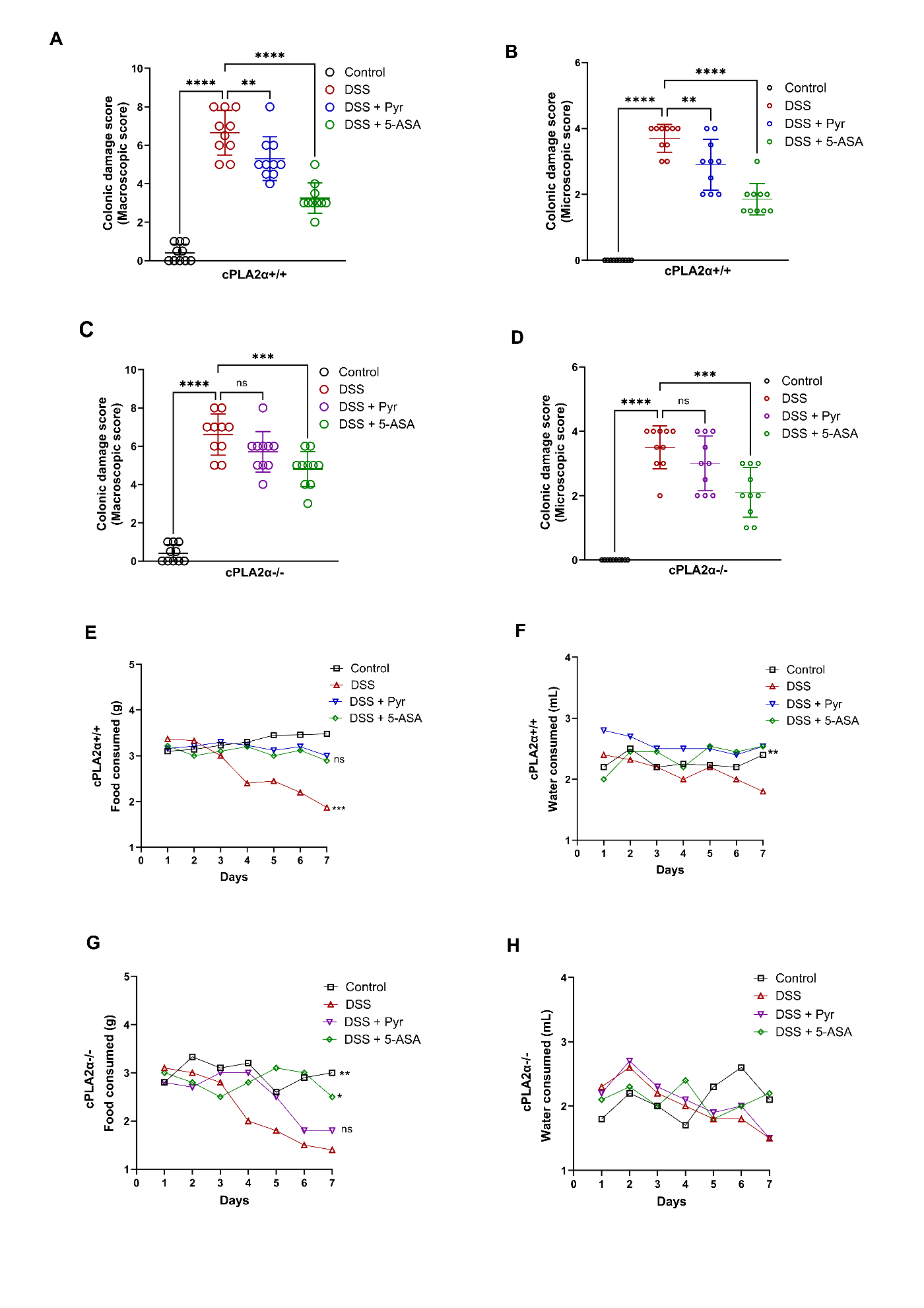


**Figure S3 (A-B)** Histopathological scoring of the colonic slides stained with H & E of cPLA2α +/+. (A) Macroscopic, (B) Microscopic. **(C-D)** Histopathological scoring of the colonic slides stained with H & E of cPLA2α -/-. (A) Macroscopic, (B) Microscopic. **(E)** Food intake and **(F)** Water consumed in cPLA2α +/+ mice. **(G)** Food intake and **(H)** Water consumed in cPLA2α -/- mice.

**Table S1:** Score parameters for disease activity index (DAI)

| **Score** | **Stool consistency** | **Rectal bleeding** |
| --- | --- | --- |
| 0 | Normal | Normal color stool |
| 1 | Mildly soft | Brown color stool |
| 2 | Very soft | Reddish color stool |
| 3 | Watery soft | Bloody loose stool |
| 4 | Diarrhea | Bloody diarrhea |

**Table S2:** Criteria for macroscopic scoring colonic damage

| **Feature** | **Score** |
| --- | --- |
| Normal appearance | 0 |
| Focal hyperemia, no ulcers | 1 |
| Ulceration without hyperemia or bowel wall thickening | 2 |
| Ulceration with inflammation at one site | 3 |
| ≥ 2 sites of ulceration and inflammation | 4 |
| ≥ 2 Major sites of damage extending > 1 cm along the length of the colon | 5 |
| Damage extended to >2 cm along the length of the colon, increase the score by one for each additional cm of damage | 6-10 |

**Table S3:** Criteria for microscopic scoring colonic damage

| **Score** | **Inflammatory cell infiltrations** |
| --- | --- |
| 0 | Normal |
| 1 | Inflammatory cells only infiltrated the mucosa |
| 2 | Inflammatory cells reach mucosa and sub-mucosa |
| 3 | Inflammatory cells were found in 75% of the intestinal wall |
| 4 | Inflammatory cells were found in the whole intestinal wall |

**Table S4:** List of mouse-specific primer sequences used for real-time PCR

| **Symbol** | **Full name** | **Forward (5′ - 3′)** | **Reverse (3′ - 5′)** |
| --- | --- | --- | --- |
| IL-1β | Interleukin-1 beta | TTCACCATGGAATCCGTGTC | GTCTTGGCCGAGGACTAAGG |
| IL-6 | Interleukin 6 | CCTCTGGTCTTCTGGAGTACC | ACTCCTTCTGTGACTCCAGC |
| TNF-α | Tumor necrosis factor-alpha | ATGAGCACAGAAAGCATGA | AGTAGACAGAAGAGCGTGGT |
| IL-17 | Interleukin-17 | TCTCCACCGCAATGAAGACC | CACACCCACCAGCATCTTCT |
| IL-23 | Interleukin-23 | GCTGTGCCTAGGAGTAGCAG | TGGCTGTTGTCCTTGAGTCC |
| Cldn2 | Claudin-2 | TATGTTGGTGCCAGCATTGT | TCATGCCCACCACAGAGATA |
| Ocln | Occludin | CCTCCAATGGCAAAGTGAAT | CTCCCCACCTGTCGTGTAGT |
| ZO-1 | Zonula occludens-1 | CCACCTCTGTCCAGCTCTTC | CACCGGAGTGATGGTTTTCT |
| MCP-1 | Monocyte Chemoattractant Protein-1 | GCTCAGCCAGATGCAGTTAA | TCTTGAGCTTGGTGACAAAAACT |
